# Supplementary figures and images for: Ancestral origins are associated with SARS-CoV-2 susceptibility and protection in a Florida patient population
Source: PLoS One. 2023 Jan 17;18(1):e0276700. doi: 10.1371/journal.pone.0276700 (PMC9844918; doi:10.1371/journal.pone.0276700)

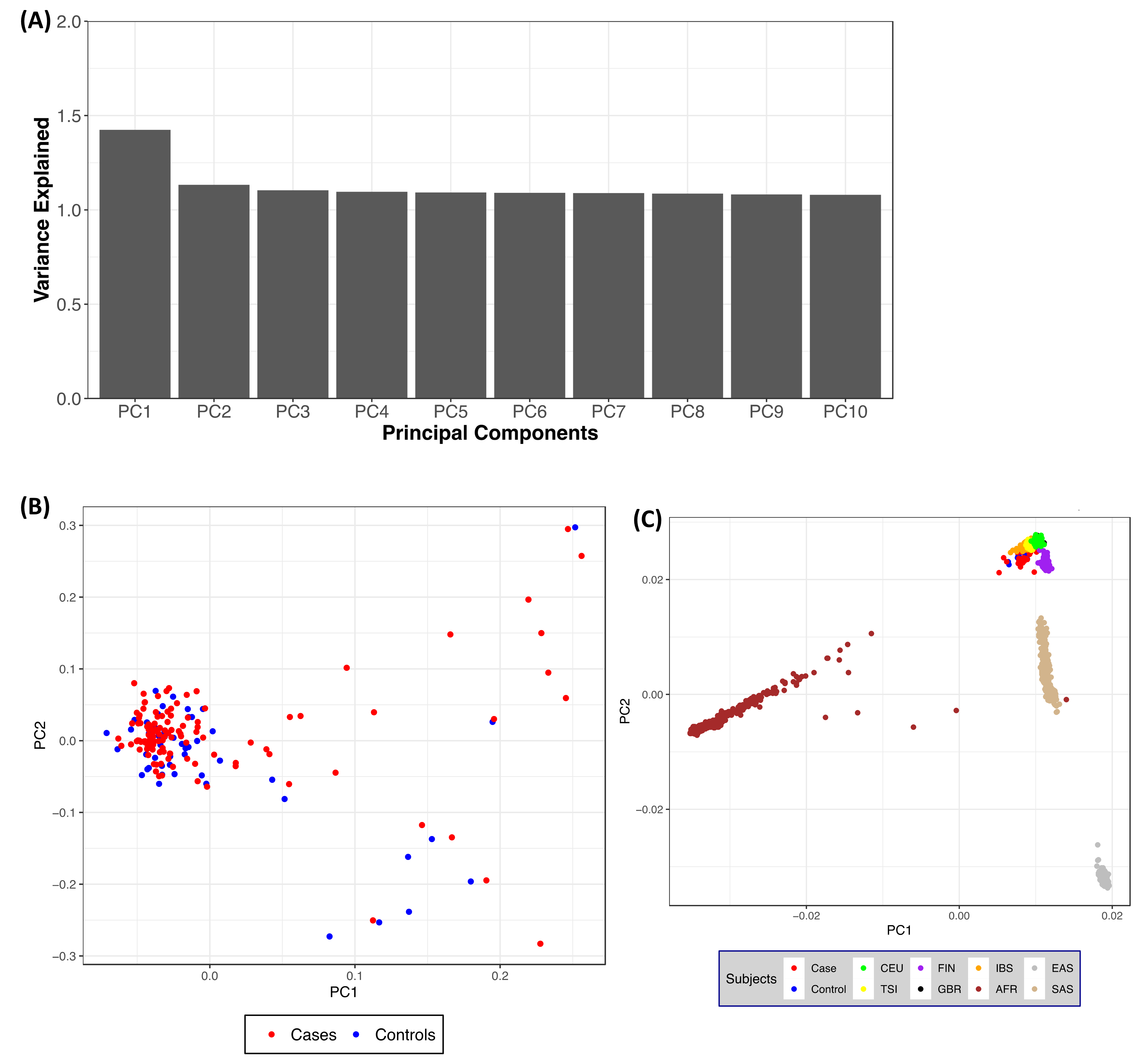

Supplement: S1 Fig — (A) Differential principal components analysis based on variance; (B) Case (red) and control (blue) samples were plotted by PC1 and PC2; (C) 1000 genome reference population was used and plotted by PC1 and PC2, case and controls samples were overlaid with the reference population to show ethnic distribution. (TIF) [file pone.0276700.s001.tif]

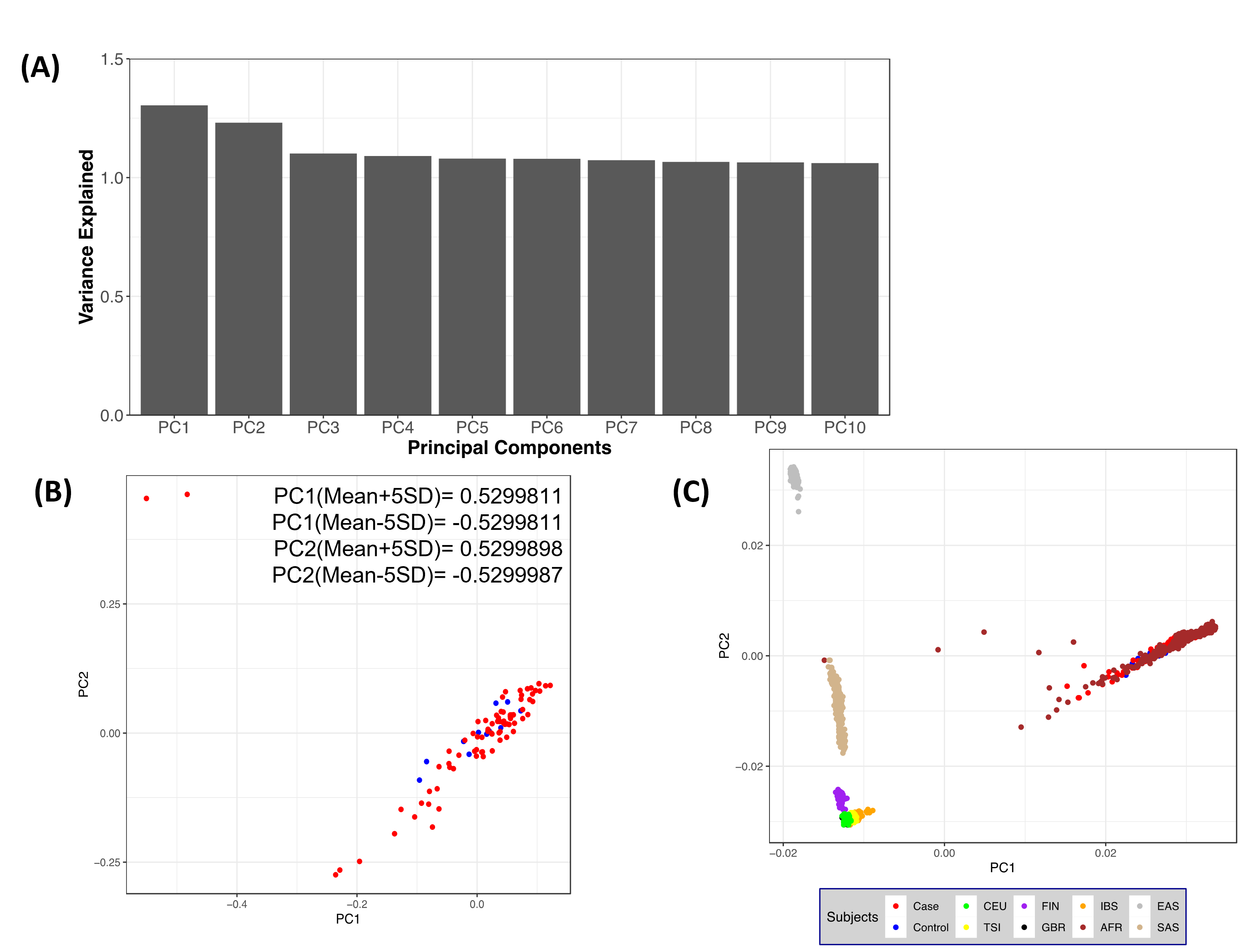

Supplement: S2 Fig — (A) Differential principal components analysis based on variance; (B) Case (red) and control (blue) samples were plotted by PC1 and PC2; (C) 1000 genome reference population was used and plotted by PC1 and PC2, case and controls samples were overlaid with the reference population to show ethnic distribution. (TIF) [file pone.0276700.s002.tif]

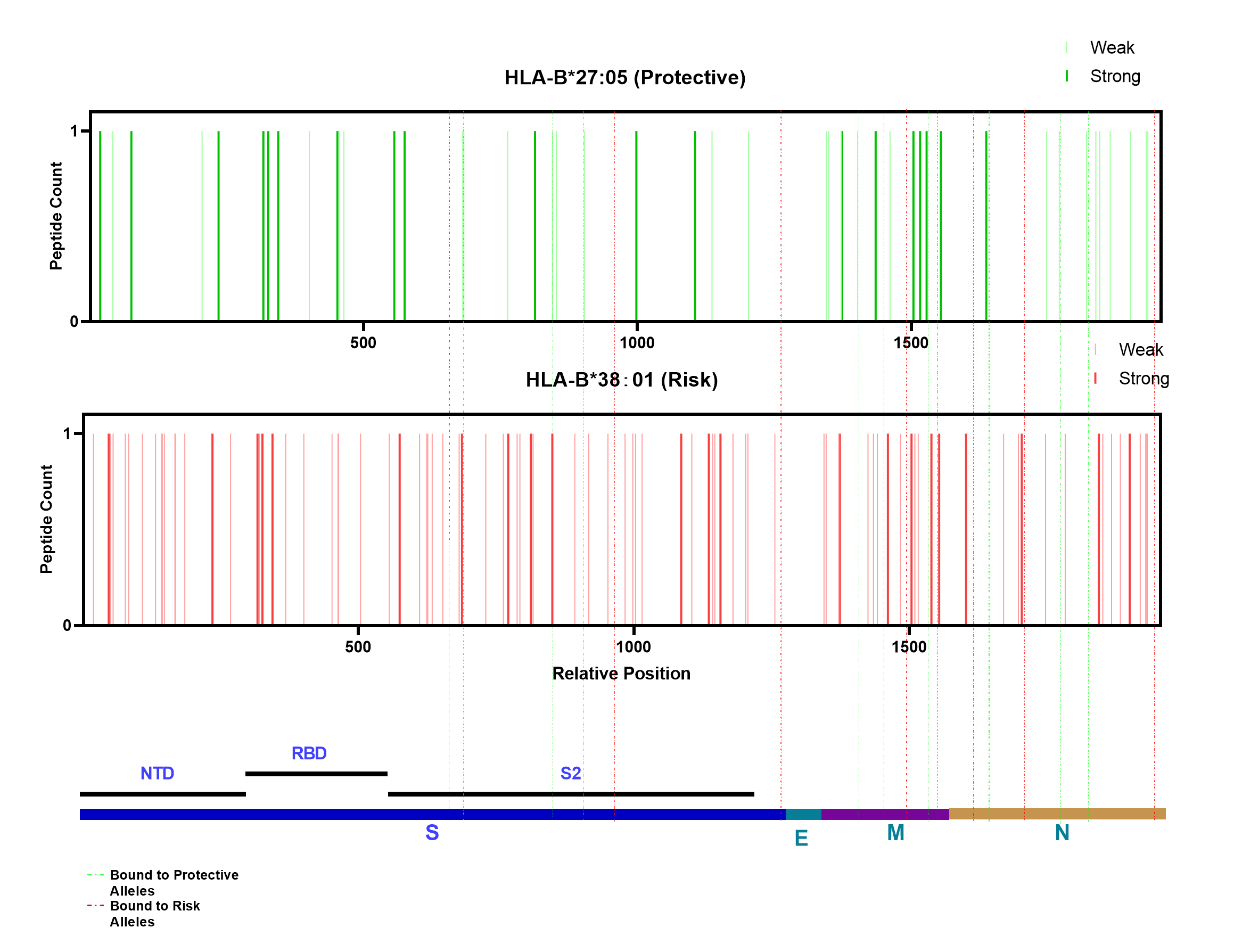

Supplement: S3 Fig — Dark and light bars indicating the identified stronger (< 0.5%Rank) and weaker (< 2%Rank) binding 9 mer peptides, respectively. With green and red indicating protective and risk alleles association, respectively. Dashed lines are the selected top three peptides in each structural protein that were presented with the greatest variation in binding affinity, marked by the final tendency of alleles (green: protective allele; red: risk allele). The relative positions are arranged by successive structural proteins in the order of S, M, N and relative lengths as indicated in the bottom. (TIF) [file pone.0276700.s003.tif]

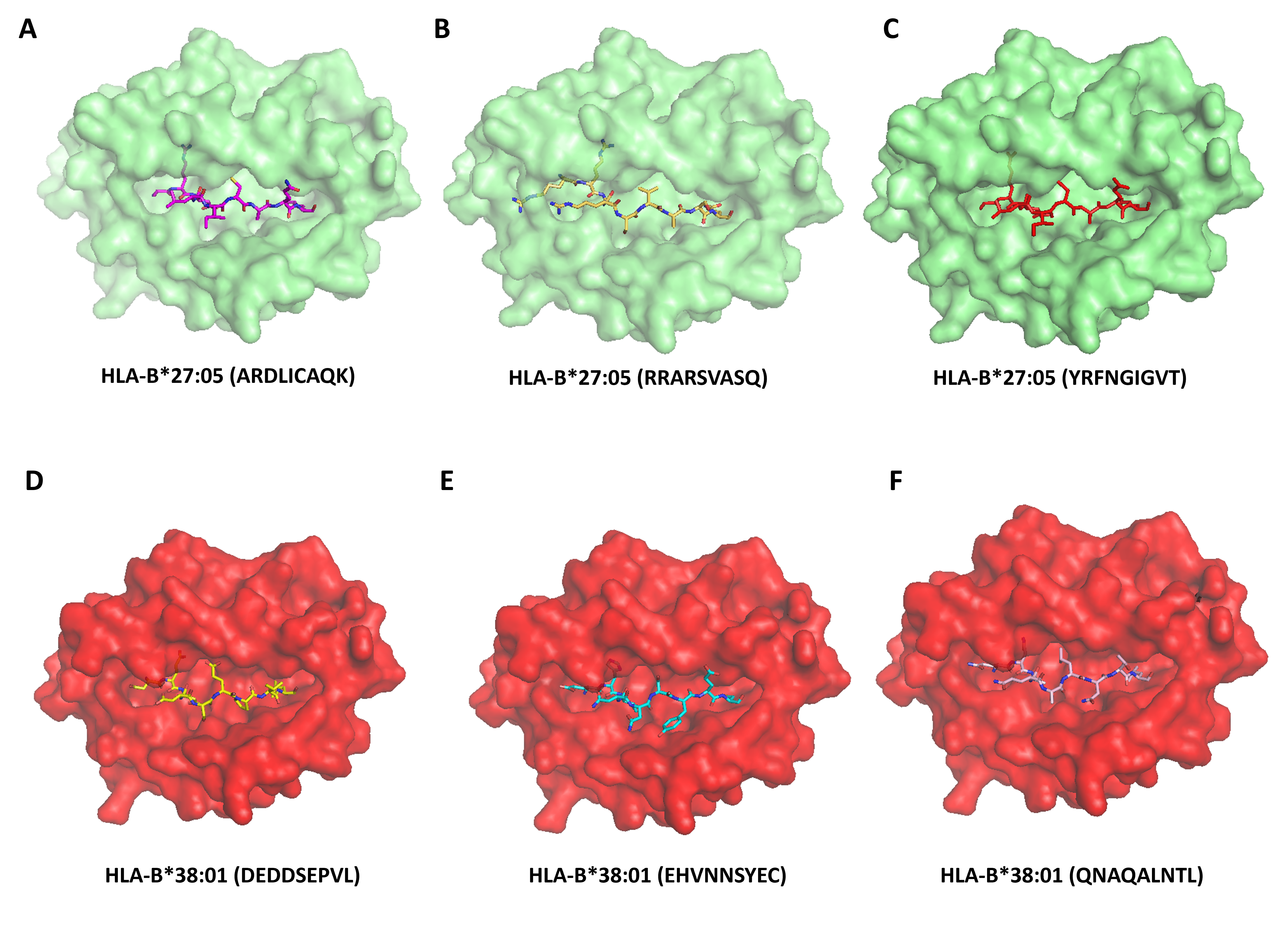

Supplement: S4 Fig — (A-C) HLA-B*27:05 binds to ARDLICAQK, RRARSVASQ and YRFNGIGVT derived from the S protein with affinity estimated Kd 1534 nM, 438.61 nM, 576.03 nM respectively. (D-F) HLA-B*38:01 binds to DEDDSEPVL, EHVNNSYEC and QNAQALNTL derived from the S protein with affinity estimated Kd 12729.29 nM, 8508.57 nM, 12297.96 nM respectively. (TIF) [file pone.0276700.s004.tif]
