# Supplementary material for: Ancestral origins are associated with SARS-CoV-2 susceptibility and protection in a Florida patient population
Source: PLoS One. 2023 Jan 17;18(1):e0276700. doi: 10.1371/journal.pone.0276700 (PMC9844918; doi:10.1371/journal.pone.0276700)
Supplement: Attachment — Submitted filename: Shen et al. Response to Reviewers.docx [file pone.0276700.s009.docx]

We would like to thank the reviewers and editorial team for your thoughtful and thorough comments on our manuscript. We have carefully considered each comment and have made changes and updates accordingly. Below is a detailed explanation of whether each change was incorporated. These changes are tracked in the revised manuscript; the number of lines marked up is based on the revised manuscript with track change.

Journal Requirements:

When submitting your revision, we need you to address these additional requirements.

1. Please ensure that your manuscript meets PLOS ONE's style requirements, including those for file naming. The PLOS ONE style templates can be found at

We have checked our manuscript and made changes to meet PLOS ONE's style requirements.

2. Please provide additional details regarding participant consent. In the ethics statement in the Methods and online submission information, please ensure that you have specified what type you obtained (for instance, written or verbal, and if verbal, how it was documented and witnessed). If your study included minors, state whether you obtained consent from parents or guardians. If the need for consent was waived by the ethics committee, please include this information.

We have added "The written informed consent was collected from each participant prior to the start of the sampling" in the Method section (line 78).

3. We note that the grant information you provided in the 'Funding Information' and 'Financial Disclosure' sections do not match.

We have corrected this issue in the resubmission.

4. Thank you for stating the following in the Acknowledgments Section of your manuscript:

"CQN is supported financially in part by PHS grants DE028544 and DE028544-02S1 from the National Institute of Dental and Craniofacial Research. We thanked Ms. Maria Cecilia Lopez from the UF Genetics Institute for performing the GWAS assays. We appreciated Dr. Patrick Concannon at the UF Genetics Institute for the insightful discussion."

We note that you have provided additional information within the Acknowledgements Section that is not currently declared in your Funding Statement. Please note that funding information should not appear in the Acknowledgments section or other areas of your manuscript. We will only publish funding information present in the Funding Statement section of the online submission form.

Please remove any funding-related text from the manuscript and let us know how you would like to update your Funding Statement. Currently, your Funding Statement reads as follows:

"The funders had no role in study design, data collection and analysis, decision to publish, or preparation of the manuscript"

Please include your amended statements within your cover letter; we will change the online submission form on your behalf.

We have deleted "CQN is supported financially in part by PHS grants DE028544 and DE028544-02S1 from the National Institute of Dental and Craniofacial Research" from the acknowledgment section. We have added the amended statement in the cover letter.

5. In your Data Availability statement, you have not specified where the minimal data set underlying the results described in your manuscript can be found. PLOS defines a study's minimal data set as the underlying data used to reach the conclusions drawn in the manuscript and any additional data required to replicate the reported study findings in their entirety. All PLOS journals require that the minimal data set be made fully available. For more information about our data policy, please see [http://journals.plos.org/plosone/s/data-availability](https://urldefense.proofpoint.com/v2/url?u=http-3A__journals.plos.org_plosone_s_data-2Davailability&d=DwQGaQ&c=sJ6xIWYx-zLMB3EPkvcnVg&r=D-W48yQhaGbDCe8D26mUbg&m=EwVYaTyqrGJrTNJmAWgZWt0Eyfbg7RgfVUu1UAdREdwOHH65DMM32BzGuzEPeC37&s=s6u0sIFfyZqx8_BMz5ncf8HPTdD0V9hTX9z5gw73eUA&e=).

Upon re-submitting your revised manuscript, please upload your study's minimal underlying data set as either Supporting Information files or to a stable, public repository and include the relevant URLs, DOIs, or accession numbers within your revised cover letter. For a list of acceptable repositories, please see [http://journals.plos.org/plosone/s/data-availability#loc-recommended-repositories](https://urldefense.proofpoint.com/v2/url?u=http-3A__journals.plos.org_plosone_s_data-2Davailability-23loc-2Drecommended-2Drepositories&d=DwQGaQ&c=sJ6xIWYx-zLMB3EPkvcnVg&r=D-W48yQhaGbDCe8D26mUbg&m=EwVYaTyqrGJrTNJmAWgZWt0Eyfbg7RgfVUu1UAdREdwOHH65DMM32BzGuzEPeC37&s=REr-KfXRJpM_2DajRZInwePoUfG_x6urpHRAmHGAeJk&e=). Any potentially identifying patient information must be fully anonymized.

Important: If there are ethical or legal restrictions to sharing your data publicly, please explain these restrictions in detail. Please see our guidelines for more information on what we consider unacceptable restrictions to publicly sharing data: [http://journals.plos.org/plosone/s/data-availability#loc-unacceptable-data-access-restrictions](https://urldefense.proofpoint.com/v2/url?u=http-3A__journals.plos.org_plosone_s_data-2Davailability-23loc-2Dunacceptable-2Ddata-2Daccess-2Drestrictions&d=DwQGaQ&c=sJ6xIWYx-zLMB3EPkvcnVg&r=D-W48yQhaGbDCe8D26mUbg&m=EwVYaTyqrGJrTNJmAWgZWt0Eyfbg7RgfVUu1UAdREdwOHH65DMM32BzGuzEPeC37&s=79M4_ucYKBXwxnPhJjRJ-TVFKCp9VpMqfnsDJoSsX5A&e=). Note that it is not acceptable for the authors to be the sole named individuals responsible for ensuring data access.

We will update your Data Availability statement to reflect the information you provide in your cover letter.

We have revised the "Data availability" with additional text "The data discussed in the study have been deposited in NCBI's Gene Expression Omnibus (GEO) and are accessible through GEO Series accession number GSE211972 (<https://www.ncbi.nlm.nih.gov/geo/query/acc.cgi?acc=GSE211972>)."

**Reviewer #1**

Specific comments:

1. Fig. 3: To map which viral epitopes are presented by the protective and risk HLAs, the authors chose HLA-C*12:03 as a risk allele in the EUR ancestry group. However, I am very wondering how important HLA-C is in the HLA class I-related antigen presentation in comparison with HLA-A and HLA-B. I think the authors should have chosen HLA-B*38:01 rather than HLA-C*12:03 because HLA-B*38:01 showed a very big odds ratio (OR = 1639.00) (Table 2).

We appreciate the reviewer's suggestion. The reviewer is correct that HLA-B*38:01 showed the highest odds ratio in the EUR ancestry. The main reason we chose HLA-C*12:03 to analyze was that HLA-C*12:03 has the lowest p-value, which we thought was more typical when considering potential molecular mechanisms in viral epitope mapping. We agree with the reviewer that HLA-B*38:01, which has a very big odds ratio, should be examined as well. As recommended, we performed the epitope mapping analysis for HLA-B*27:05 (protective) and HLA-B*38:01 (risk) (see S3 and S4 Figs and S3 table for details). With the new data, we revised the result section by adding the following sentences (lines 379-384).

"Considering that HLA-B*38:01 has a large odds ratio (OR = 1639.00), we also did the same analysis **(S3 Table) (S3 and S4 Figs)** with HLA-B*38:01 as a risk allele compared to HLA-B*27:05 (protective). There was no obvious difference in the pattern and type of antigen presentation preference between the two alleles, as the presentation of S proteins was concentrated in the S2 region, and both alleles were unable to present E proteins."

2. Page 19, lines 1-3 & Fig. 7B: The HLA-B*38:1 is not likely to show a positive or upward trend between the increase in the allele frequencies and rise in mortality (p=0.6092) (Figure 7B).

We thank the reviewer for pointing out the inaccuracy of this description. We have made the following changes in lines 510-511.

"Although the trends were similar, this was not significant in the mortality correlation (p=0.6092) **(Fig 7B)**".

3. Page 22, line 2 & Page 23, line 10: "the top three alleles" should be "the top three peptides"?

We thank the reviewer for pointing out this wording error. We checked and switched "alleles" to "peptides" in lines 121, 126, and 360.

4. Page 23, line 5 & page 42, line 8, & Fig. 5: HLA- DQA1*05:01 is unlikely to be a risk allele because this allele is not found in Table 3.

Thanks for pointing out our confusing description. The reviewer is correct that HLA- DQA1*05:01 is indeed not a significant risk allele in AFR group.

According to the NetMHCIIpan 4.0 server (<https://services.healthtech.dtu.dk/service.php?NetMHCIIpan-4.0>), the prediction based on HLA-DQ molecules requires both α and β chains, and the only significant allele in the AFR group is HLA- DQB1*03 :01 (β chains). To prevent misleading random selection, we chose HLA- DQA1*05:01 as the α-strand for prediction. The detailed reasons are as follows.

1. According to the allele frequency network database (http://allelefrequencies.net/default.asp), the haplotype frequency of HLA-DQA1*05:01-DQB1*03:01 is relatively high in the population (up to 34%).
2. HLA-DQA1*05:01 does appeared in our HLA imputation with OR = 2.555, and p = 0.2915 (not significant)
3. HLA-DQA1*05:01-DQB1*03:01 belongs to the HighQ-DQ datasthe et in NetMHCIIpan 4.0 server, which possesses high accuracy prediction of binding patterns.

To make it clear, we revised the result (lines 389-391) by adding: "Prediction based on HLA-DQ molecules requires both α and β chains, we chose HLA- DQA1*05:01 (not significant in HLA estimation) as the α-chain based on the overall haplotype frequency and predictive accuracy."

**Reviewer #2:**

1. The determination of ancestry is not well defined, and supporting information was not available for review. The QC of the dataset was used "as previously described" but no ref is given and the information within the available methods is not sufficient. This needs to be described in more detail.

We apologize for not making these details clear in the main text. The process of determining ancestry and the quality control steps were written in the methods section, namely "Assessment of population stratification" and "Quality control” sections. The determination of ancestry by using genome reference SNP is a relatively standardized process in GWAS. We did not stratify the patient populations based on the base on the information provided by participants.

As recommended by the reviewer, references and new descriptions were added to the manuscript to clarify the methodology. It can be found in lines 158-159, "Due to the ethnically diverse nature of Florida residents, we performed quality control of the dataset as described previously in methods[21]."

2. With a small sample size of 284 subjects, and no significant alleles determined for the AFR ancestry, it may be better to analyze the cohort as a whole, or breakdown between hospitalized and non-hospitalized subjects.

We agree with the reviewer that it is better to analyze the cohort as a whole. In fact, that was what we did when we first conducted the study. However, when we performed the quality control step principal component analysis, it showed a significant difference in PC1. Furthermore, when we mapped our samples to the 1000 genomic reference population, we saw two independent clusters on PCA, and a larger number of sample scatters that could not be genetically annotated (please see the accompanying figure below). This means that our samples are fundamentally different in terms of genetic background. Some of the previous studies we cited, such as [33], used the same method as we did to exclude non-European ancestry and even subdivided the population to 8,036 Spanish and 4,273 Italian. Given the diversity of the Florida population, we decided to classify by genetic background for the accuracy of our study, even though our sample size was small.

Left, Case (red) and control (blue) samples were plotted by PC1 and PC2; Right, 1000 genome reference population was used and plotted by PC1 and PC2, case and controls samples were overlaid with the reference population to show ethnic distribution.

3. The breakdown of ancestry to look at susceptibility to COVID-19 within this paper only considers the genetic component as analyzed and does not fully consider or discuss the social impacts that can impact disease exposure or severity, etc. These include, socio-economic status, health care access, education level etc. To make inferences based on ethnicity alone is questionable without fully addressing other contributing factors. This information may not be available for the studied cohort but should be considered when making conclusions about COVID-19 susceptibility and discussed further in the manuscript. The overall language used to compare different ancestries for COVID-19 susceptibility or severity needs to be carefully chosen and justified.

We appreciate the reviewer's thoughtful insight. As recommended, we have revised the discussion with new references to address this issue.

Lines 634-681: "Age and pre-existing medical conditions such as hypertension, obesity, chronic lung disease, diabetes mellitus, and cardiovascular disease are associated with the severity and hospitalization rates of COVID-19 patients. In the COVID-NET catchment population which represents approximately 10% of the U.S. population with an equal ratio of male and female, 54% of COVID-19-associated hospitalizations occurred in males and 46% occurred in females[1]. These data suggest that males may be disproportionately affected by COVID-19 compared with females. Additionally, 59% of residents are white, 18% are black, and 14% are Hispanic; however, among 580 hospitalized COVID-19 patients with race/ethnicity data, approximately 45% were white, 33% were black, and 8% were Hispanic[1], suggesting that black populations might be unevenly affected by COVID-19[4,7]. Social economics and access to quality healthcare are important factors in clinical outcomes. The epidemiology and the underlying reasons account for these differences need further investigation and beyond the scope of this study. Our data showed that the mean age and gender distribution of the EUR and AFR ancestry did not significantly differ in the case or control group. In conclusion, this study provides the first insight of group analysis regarding the effects of SARS-CoV-2 infection in different ancestry origins from the same region, including the susceptibility and disease severity. Due to the limited sample size, further validation is needed by *in-vitro* experiments. This study demonstrated that specific protective or less protective HLA alleles are predicted to present viral antigens differently, which could impact and contribute to the susceptibility and clinical outcome among populations."

4. The authors state that they determined mild vs severe cases based on whether a nasal swab or tracheal aspirates, however this information only captures disease at the collection time. Do the authors a way to determine if the subjects that provided nasal swabs remained "mild" (ie no hospitalization")?

This is an important point that the reviewer raised. In response to this comment, during the past two months, we have revised our IRB to determine the prospective clinical status of these donors. Furthermore, we needed to identify the comorbidities (in response to comment #5). This information would provide additional important insights into the study. Unfortunately, as quoted by the Biorepository, "No information on the setting where samples were collected was available. As a consequence, further analyses on patient hospitalization status, length of stay, mortality, and comorbidities were not possible".

5. In addition, a table with more information about each subject within the different ancestries would also be helpful. Are those in one cohort older or younger than the other? Do they have any comorbidities, especially subjects within the hospitalization group? Were any of the subjects vaccinated against COVID-19?

We appreciate the reviewer's feedback. Per suggestion, Table S4 was created with all the available information provided to us and added to the manuscript as supporting information. We have revised the discussion with this information (lines 641-642). Regarding vaccination status, all control and positive samples were collected before the COVID-19 vaccine was available, and theoretically, they were not vaccinated. Regarding the comorbidities and hospitalization status, please see our previous response (comment #4).
