# Supplementary material for: Ancestral origins are associated with SARS-CoV-2 susceptibility and protection in a Florida patient population
Source: PLoS One. 2023 Jan 17;18(1):e0276700. doi: 10.1371/journal.pone.0276700 (PMC9844918; doi:10.1371/journal.pone.0276700)
Supplement: Attachment — Submitted filename: Response to Reviewers.docx [file pone.0276700.s010.docx]

We would like to thank the editor and reviewer again for the feedback. We have carefully considered your comments. Below is a detailed explanation of whether each change was incorporated. Additionally, we have made one correction regarding the number of peptides we screened with HLA-C*12:03 (changed from 46 to 151 peptides, line 271). Lastly, we have corrected a few grammatical errors in the revised manuscript.

Reviewer #2: The authors have submitted a well revised manuscript that more clearly communicates their data. However, upon revising the submitted material, it was noted that the AFR control group only consisted of 11 subjects, while the EUR control group comprised 57 (Supp Table 4). Can the authors comment on why there are so few individuals in the ARF control group compared to the EUR group and does this affect any of the conclusions made about the data?

We appreciate the reviewer's concern. We sampled based on whether or not being diagnosed with COVID-19 and disease severity. The distribution in the control group matched the population distribution in Florida, as we discussed in the result section with the heading "GWAS analysis of COVID-19 patients with European and African ancestries", in which 53% of Floridians are White (Non-Hispanic), with 21.6% being White (Hispanic), Black or African American (Non-Hispanic) (15.2%). As presented, the case group had a slightly higher proportion of AFRs, which is consistent with our conclusion of potential genetic susceptibility. Since we took EUR and AFR separately for comparison with their respective control group, the impact on the result should be minimal. Further *in vitro* experiments are needed to confirm these findings as proposed in the discussion.

Also to note, the Supplemental tables file names are mislabeled.

We thank the reviewer for pointing out the mislabeled file names. We have corrected the file names for all three supplementary tables.
